# The Role of Curcumin in the Modulation of Ageing

**DOI:** 10.3390/ijms20051239

**Published:** 2019-03-12

**Authors:** Anna Bielak-Zmijewska, Wioleta Grabowska, Agata Ciolko, Agnieszka Bojko, Grażyna Mosieniak, Łukasz Bijoch, Ewa Sikora

**Affiliations:** Nencki Institute of Experimental Biology, Polish Academy of Sciences, 3 Pasteur St., 02-093 Warsaw, Poland; w.grabowska@nencki.gov.pl (W.G.); a.ciolko@nencki.gov.pl (A.C.); a.bojko@nencki.gov.pl (A.B.); g.mosieniak@nencki.gov.pl (G.M.); l.bijoch@nencki.gov.pl (Ł.B.)

**Keywords:** ageing, anti-cancer, autophagy, microbiota, senescence, senolytics

## Abstract

It is believed that postponing ageing is more effective and less expensive than the treatment of particular age-related diseases. Compounds which could delay symptoms of ageing, especially natural products present in a daily diet, are intensively studied. One of them is curcumin. It causes the elongation of the lifespan of model organisms, alleviates ageing symptoms and postpones the progression of age-related diseases in which cellular senescence is directly involved. It has been demonstrated that the elimination of senescent cells significantly improves the quality of life of mice. There is a continuous search for compounds, named senolytic drugs, that selectively eliminate senescent cells from organisms. In this paper, we endeavor to review the current knowledge about the anti-ageing role of curcumin and discuss its senolytic potential.

## 1. Introduction

Demographic data unquestionably show that the population of elderly and very elderly people is continuously increasing. The population of people aged 65 and above represents 8.7% of the total population. However, this percentage differs between continents and is around 15–16% in North America, Europe and Central Asia, but only about 5% in the Middle East, North Africa and South Asia [1]. The increase of lifespan is not really satisfactory without an improvement of healthspan. We would like to live longer, but in good health, which is necessary to enjoy the world around us. Actually, there is a great deal of evidence that the ageing process is malleable and the rate and quality of ageing can be modulated [2]. In order to be able to postpone ageing, it is urgent to reveal the mechanisms of ageing.

It is commonly accepted that cellular senescence plays a very important role in organismal ageing and age-related diseases [3]. Namely, it has been observed that senescent cells accumulate in the tissues and organs of old animals and humans, and that proliferation potential differs among cells derived from individuals of different age [4,5,6,7,8]. Even though the actual number of senescent cells seems not to be very high and fluctuates between a few and a dozen percent, changes in the extracellular milieu caused by the increased production of cytokines by senescent cells, and the senescence-associated impairment of regenerative processes, can lead to spectacular organismal dysfunctions. Moreover, senescent cells contribute to the onset and progression of diseases, the frequency of which increases with age. The accumulation of senescent cells has been observed in the course of almost all age-related disorders [9]. Breakthrough experiments, which have definitely proved the involvement of cell senescence in the progression of ageing and age-related diseases, came from animal studies. It has been clearly shown that the elimination of senescent cells alleviated the symptoms of ageing and age-related disorders and improved the quality of life of genetically modified animals [10,11]. A recently proposed strategy is to protect people from ageing instead of curing particular diseases [12], and to go deeper, to eliminate cell senescence in order to prevent ageing dysfunctions. In animal models, some progress in postponing ageing has been achieved, but certain approaches cannot be transferred to humans because of the potential detrimental effects of the long-term application of anti-ageing agents (not to mention genetic manipulations). The best approach to ageing protection cannot be demanding, should be easily available, lack any risk of side effects, and should be inscribed in our lifestyle as diet or physical activity. Much hope is currently placed in natural compounds, and some promising results have already been obtained. One of such compounds is a polyphenol: curcumin. Curcumin’s role in postponing ageing in animal models has already been documented, but certain data in humans must be verified in longitudinal trials.

## 2. Cellular Senescence

Cellular senescence was described for the first time about 60 years ago by Leonard Hayflick and Paul Moorhead [13]. Since that time, a concerted effort has been undertaken to explore the role and mechanisms of this fundamental cellular process. The role of senescence is complex and depends on the age of the organism [2]. In a young organism, cell senescence serves a beneficial function. Namely, it is essential in embryonic development (senescent cells are eliminated by immune cells as an element of body shaping), in tissue regeneration and as a cancer barrier (senescent cells are not able to proliferate). In an old organism, the number of senescent cells increases, and they generate a state of low chronic inflammation, via so called senescence-associated secretory phenotype (SASP), produce excessive reactive oxygen species (ROS) and cause microenvironmental changes, which support tumor progression. Generally, cell senescence is detrimental due to the role of senescent cells in ageing and age-related diseases. On the other hand, the role of cell senescence in organismal homeostasis should not be neglected. Moreover, senescence is a highly dynamic process induced by genetic and epigenetic changes [14]. During the lifetime of an organism, cells experience several types of intrinsic (metabolic functions with ROS production and DNA replication) and extrinsic (chemical and physical genotoxic events) stresses. Following DNA damage, cells repair their DNA to eliminate the possibility of mutations that can provoke neoplastic transformation. Cell response to a specific stress implies correct DNA repair to completely recover damaged cells or, alternatively, cells harboring unrepairable damages may enter apoptosis or senescence [15].

The most important feature of senescence is the cessation of proliferation (it concerns proliferation-competent cells), increased level of cell-cycle inhibitors (p21, p16), increased activity of a lysosomal enzyme, senescence-associated β-galactosidase (SA-β-gal), increased number of DNA double-strand breaks (DSBs) and activation of the DNA damage response (DDR) pathway, along with changes in chromatin structure due to modified gene expression and higher vulnerability to DNA damage [16]. One of the most important features of senescent cells is the appearance of senescence-associated secretory phenotype (SASP). SASP arises due to the increased production and secretion of proteins, which can act both in a paracrine and autocrine manner and are involved in the generation of low-grade inflammation. This exerts a bystander effect; that is, it induces senescence in neighboring cells. Cells can undergo senescence as a result of telomere erosion (replicative senescence) or in response to some external (chemical and physical factors) or internal (oncogene overexpression, increased ROS production, DNA damage, ER-stress, and chromatin structure dysfunction) stimuli. The latter form of senescence is termed stress-induced senescence (SIPS, stress-induced premature senescence) [17]. It seems that senescent cells are very well characterized and relatively easy to distinguish from non-senescent cells [18]. On the other hand, the sharp definition of what is cell senescence, that used to exist in the past, is not anymore so straightforward. There is a growing body of evidence showing that certain features of cell senescence and mechanisms of its induction can differ depending on the cell and senescence type [19]. This concerns, for example, different propensity of cell to respond to stress [15], some metabolic differences between stress-induced and replicative senescence [14,20] or differences in SASP components [21]. An interesting question is whether post-mitotic cells can undergo cell senescence [22], and if cancer cell senescence is reversible; in other words, whether SA-β-gal activity in these cells is indicative of their senescence or is simply unspecific [23]. Despite this dilemma, senescent cells simply identified on the basis of high level of cell cycle inhibitors, can be eliminated from a murine body leading to its rejuvenation (see [24], chapter 5.5).

## 3. Senescence and Age-Related Diseases

Senescent cells are linked with many age-related diseases., such as neurodegenerative diseases (Alzheimer’s and Parkinson’s disease; AD and PD, respectively), cataract, glaucoma, cardiovascular diseases (CVD, atherosclerosis and hypertension), chronic obstructive pulmonary disease (COPD), idiopathic pulmonary fibrosis (IPF), diabetes type II, sarcopenia, osteoarthritis, osteoporosis and certain types of tumors [9,12,25,26,27,28,29,30,31,32].

## 4. Anti-Ageing Intervention

The development of science and medicine has contributed to the increase in human lifespan. However, with the increase in life expectancy, an increase in the incidence of age-related diseases is observed. Therefore, it is crucial to find an approach to prevent or delay ageing and the onset of age-related diseases. Animal studies have provided us with a wealth of knowledge and cues, and several strategies to elongate the lifespan of model animals, mainly based on genetic manipulation, have been established. So far, the only non-genetic approach that can extend longevity is dietary/calorie restriction (DR/CR) [33,34]. This intervention involves a 20-40% reduction in calorie intake without causing malnutrition and has been shown to be effective in several species including yeast, fruit flies, nematodes, rats, dogs [35,36,37] and even primates [38]. Moreover, epidemiological studies support the positive effects of CR in humans. For example, on a Japanese island of Okinawa, the number of centenarians (people over 100 years of age) is five times higher than in any other part of the world. A study revealed that the mean calorie consumption of adult Okinawans is 17% lower than that of an average Japanese adult and 40% lower than that of an average adult citizen of the United States [39,40]. The effects of calorie restriction are not limited to lifespan extension but include also improved cardiovascular and metabolic health, decreased incidence of cancer, along with attenuated neurodegeneration and sarcopenia [39,41,42]. As sticking to a CR regime can be uncomfortable, scientists are looking for drugs, supplements or less drastic dietary intervention that could mimic CR. One of such interventions is intermittent fasting (IF), which has recently been gaining increased attention [43,44]. Goodrick’s studies on rats maintained on alternative-day fasting regimen showed that, depending on the age at which the diet was started, the animals lived from 30% to 100% longer than animals fed ad libitum [45,46]. In turn, a recent study by Catterson et al. demonstrated that IF (2-day fed:5-day fasted) can extend the life of a fruit fly by 10% [47]. It has been proposed that CR acts by upregulating autophagy, as the inhibition of this process decreases the anti-ageing effects of this diet intervention [48] (see paragraph 5.3). Another research work suggests that the effects of CR are mediated by sirtuins, the expression of which increases as a result of such restriction [49]. Sirtuins are crucial for metabolism and are involved in cellular response to a variety of stresses such as oxidative or genotoxic stress. A decreased level or activity of these enzymes shortens the lifespan of different model organisms, while increased activity/level can improve both lifespan and healthspan [reviewed in 37]. There are a number of natural (quercetin, butein, curcumin, fisetin, kaempferol, catechins) [37,50] as well as synthetic [51] compounds that can induce sirtuin expression, enhancing lifespan and ameliorating age-related diseases. Besides diet intervention, mild physical activity has also been shown to improve health and lifespan. It is believed that low-intensity exercise can serve as a mild stressor, which activates stress response, preparing the organism to a greater threat. This activates antioxidant enzymes that reduce oxidative stress and can also activate sirtuins [52,53]. In summary, the potential and promising anti-ageing approaches in humans are related to the limitation of the amount of consumed food and/or the application of certain compounds or physical activity that mimic diet restriction.

## 5. Curcumin

Curcumin is a promising anti-ageing compound which is easily available and easy to apply in the diet, as well as being safe and not expensive. Curcumin is a widely studied nutraceutical, belonging to polyphenols, acquired from the rhizome of a plant *Curcuma longa* (turmeric), a member of the ginger family. Turmeric contains 12 active components [54], thus the percentage of curcumin (chemically known as diferuloylmethane) per dry weight of turmeric powder is no more than 3.14% [55]. Curcumin is commonly used as a spice (curry, turmeric) and yellow food dye (E100); therefore, it is consumed on a daily basis. However, curcumin is poorly absorbed by intestinal cells (low aqueous solubility and stability), rapidly metabolized by the liver (generation of less active curcumin glucuronides, see paragraph 5.6), and rapidly eliminated from an organism [56]. The highest achieved serum level of curcumin was about 1.77 µM, 1 h after administration, during the oral ingestion of 8 g of curcumin per day, or even 3.6 µM if such a dose was consumed for 3 months [57]. The Hindu people, who are the nation with the highest daily intake of curcumin (present in the turmeric spice), consume up to 100 mg/day of the active substance [58]. Due to curcumin’s limited bioavailability, the potential therapeutic use of that polyphenol might be questionable. However, numerous studies have been performed to readdress these crucial issues. Moreover, numerous attempts have been made to enhance curcumin ingestion, such as the co-administration of curcumin with piperine, the active substance from pepper, which can increase curcumin’s level in the blood by as much as 30 times [59]. This can also be achieved by increasing its aqueous solubility and stability by conjugation with alginate [60], improving cell targeting by self-assembling peptide nanofiber carrier [61] or creating curcumin-phospholipid complexes, microemulsions, liposomes, polymeric micelles and curcumin nanoparticles [56]. Furthermore, clinical trials showed that even extremely high daily doses of curcumin intake (12 g/day) were harmless to patients [57].

Curcumin, like other polyphenols, possesses pleiotropic activity (curcumin belongs to the PAINS, pan-assay interference compounds), which is considered as a serious disadvantage of natural compounds [62]. Indeed, due to its ability to interact simultaneously with many receptors (e.g., EGFR, CXCR4), growth factors (e.g., EGF, TGFβ), kinases (e.g., MAPK, FAK), transcription factors (e.g., NF-κβ, STAT1-5), enzymes (e.g., DNA pol, COX2), adhesion molecules (e.g., ICAM-1, VCAM-1), apoptotic regulators (e.g., survivin, Bcl-2), proinflammatory cytokines (e.g., interleukin (IL)-8, tumor necrosis factor (TNF)) and other proteins (e.g., p53, cyclin B1) [63,64], curcumin can evoke a broad cellular response to external stimuli. Furthermore, curcumin up- and down-regulates different kinds of miRNA [65] and takes part in epigenetic changes by inhibiting DNA methyltransferases and regulating histone modifications via effects on histone acetyltransferases and histone deacetylases [66,67,68]. However, in our opinion, this is not a disadvantage but, quite to the contrary, an advantage when one compound can affect diverse biological processes, such as the redox state, inflammation, proliferation, migration, apoptosis, wound healing and as a consequence positively affect memory, postpone ageing and age-related diseases such as atherosclerosis [63,64]. Therefore, complex, multigenic, chronic, civilization- and age-related diseases, which occur due to perturbations in multiple signaling pathways, seem to be a suitable target of curcumin-based therapy [63,64]. Due to all those properties of curcumin, it has been used in a vast amount of clinical trials as a drug or adjuvant in the treatment of various diseases [69]. Furthermore, the beneficial or detrimental effect of curcumin depends on its concentration. This phenomenon is widely described as a hormetic effect [70]: it acts as a stimulant at low and an inhibitor at high concentration. This also applies to curcumin function: in low doses, curcumin could act as a protective agent, whereas in high doses it could act as a cytostatic, cytotoxic and genotoxic agent.

### 5.1. Curcumin and Its Anti-Ageing Role

As ageing is characterized by chronic low-grade inflammation [71], polyphenol-rich foods, which have anti-inflammatory as well as antioxidant properties, can mitigate symptoms of ageing. There are plenty of examples to support the possible anti-ageing role of curcumin [72,73,74,75]. Curcumin supplementation in a diet extended the lifespan of fruit flies, nematodes and mice [76,77,78,79]. Moreover, in clinical trials, curcumin was proven to reduce symptoms of some age-related diseases such as atherosclerosis, diabetes and cancer [80,81]. It also serves as a neuroprotective agent [82]. Curcumin has also been shown to protect against chemotherapy-induced side effects such as cardiotoxicity elicited by doxorubicin [83] and radiation-induced dermatitis in breast cancer patients [84]. On the cellular level, curcumin protected HUVEC against peroxide-induced senescence [85], while a curcumin analogue, bis-demetoxycurcumin, inhibited the oxidative stress-induced senescence of WI38 fibroblasts [86]. Moreover, curcumin increased the ability of human epidermal keratinocytes to differentiate during replicative senescence [87]. There are some rationales suggesting that the anti-ageing function of curcumin is due to its ability to postpone cellular senescence. However, our recent results excluded such a possibility, at least for cells building the vasculature [88]. Curcumin did not postpone the replicative senescence of vascular smooth muscle cells (VSMC) and endothelial cells (EC) or the doxorubicin-induced senescence of VSMC [88]. Even though curcumin at low concentration (0.1–1.0 µM) was not able to postpone replicative senescence or protect cells from doxorubicin-induced senescence, it increased the level of sirtuins and AMPK in VSMC undergoing replicative senescence [88]. Therefore, it is possible that positive effects of curcumin supplementation, observed on the organismal level, can be attributed to sirtuin and AMPK induction rather than the inhibition of cellular senescence [88]. Others observed that curcumin supplementation in mice and rats enhanced the effect of exercise, affected the time of exhaustion and prevented fatigue, which was associated with an increased level/activity of AMPK and sirtuin 1 in muscles [89,90]. It has been shown that Sirt2 is indispensable for curcumin-induced *Caenorhabditis elegans* lifespan elongation [76].

In summary, curcumin is involved in the regulation of nutrient-sensing signaling pathways (impact on sirtuins, AMPK), and thus it is able to mimic caloric/diet restriction and increase the benefits coming from mild physical activity [37].

We have also tested concentrations of curcumin (5–7.5 µM for VSMC and 2.5–5 µM EC) which were close to those observed in serum after diet supplementation. Unexpectedly, both VSMC and EC underwent senescence upon such treatment [91]. Senescence induced by curcumin was DNA damage-independent and resulted from influencing many signaling pathways. The initial changes concerned decreased levels of sirtuins and AMPK, which suggests that the reduction of these proteins could be important for senescence induction (submitted). Altogether, these results show that curcumin, although is not able to postpone senescence per se, and can even induce it, may exert its anti-ageing effect via the ability to change the levels of proteins involved in the process of ageing (sirtuins, AMPK).

Moreover, it cannot be excluded that cell senescence induced by curcumin plays a beneficial role. Such a positive function has been shown in curcumin-senescent cancer-associated fibroblasts (CAF), which reduced the malignance of the tumor [92], and in hepatic stellate cells (HSC), where curcumin-induced senescence protected against liver fibrosis [93].

Another important but not always direct anti-ageing function of curcumin is its anti-tumor activity [94] (detailed description in Section 5.4). Ageing is one of the most important risk factors in some types of tumor [95]. Elderly woman and men are four-fold and seven-fold, respectively, more prone to all types of cancer than their younger counterparts. Among elderly men, cancer of the prostate, lung and colon make up around half of all diagnosed cancers. The corresponding most frequent cancers among elderly women, making up 48% of all malignant cancers, are breast, colon, lung and stomach cancer. Curcumin can protect against tumorigenesis (e.g., by protection against the toxicity of some factors present in the environment or applied during therapy), reduce cancer cell number (by the induction of cancer cell apoptosis) and inhibit metastasis (anti-angiogenic properties) [72]. Some data even suggest that cancer cells are more sensitive to curcumin than normal ones. This can be explained by taking several factors into account. The first one is related to the rate of cell proliferation. Cancer cells divide more frequently than normal ones and curcumin disturbs mitosis. This is related, among other, to the impairment of the mitotic spindle [96,97], inhibition of cdk1 kinase [94] and inhibition of sirtuin 7 [98]. The second mechanism is related to curcumin’s ability to inhibit NF-κB transcription factor, which is highly expressed in cancer cells. This property is due to the inhibition of IκB-IKK and also to the activation of sirtuin 1 and 6 [37], which inactivate, by deacetylation (sirtuin 1) or indirect interaction (sirtuin 6), RelA/p65, one of the NF-κB components. Yet another factor is associated with the increased activity of β-glucuronidase (responsible for deconiugation of glucuronides) and lower activity of UDP-glucuronosyltransferases (UGT) (an enzyme involved in glucuronides formation) in the tumor tissue [99]. This could lead to an increased local concentration of free, earlier glucuronized compounds, and increase the efficacy of apoptosis. Such action is also ascribed to curcumin (see also Section 5.6) and may improve its anti-cancer activity.

One of the considered strategies dedicated to tumor elimination is cell senescence induction. Curcumin is able to induce cellular senescence in cancer cells. This, which can be harmful for normal cells, could be beneficial in the context of cancer cells. Such an approach, with time, appears controversial, because some data have shown that the senescence of tumor cells can be reversible and can lead to cancer relapse [100].

### 5.2. Curcumin and SASP

One of the roles of curcumin in the alleviation of ageing is reduction of inflammation. Senescent cells, despite being in a non-proliferating state, remain alive, metabolically active and can influence their microenvironment. One of the most important features of senescent cells is their ability to secrete a number of proteins: mainly interleukins, chemokines and other pro-inflammatory cytokines, proteases, metalloproteinases and growth factors. This phenomenon, known as SASP, is involved in many processes such as inflammation, angiogenesis, extracellular matrix reorganization, the stimulation of proliferation and the modulation of the immune system. Depending on the cellular context, this can either be beneficial or detrimental. One of the positive aspects of SASP is that some of the secreted cytokines, such as interleukin-6 (IL-6) or interleukin-8 (IL-8), are essential in the process of inducing, maintaining and reinforcing senescence in an autocrine manner [101]. Moreover, SASP can be used as a form of communication with immune cells. Senescent cells, by secreting different chemokines (e.g., RANTES, GROα, MCP-1), may attract distinct subsets of NK cells, monocytes/macrophages, neutrophils, B cells and T cells. In consequence, these immune cells ensure the surveillance and clearance of damaged, senescent and dysfunctional cells [17,102]. This is one of the necessary steps for tissue regeneration and protection from fibrosis [103]. On the other hand, proteins secreted by senescent cells may create a tumor-promoting environment, enhance the migration of tumor cells and thus the formation of metastases, and also may induce senescence in neighboring normal cells. In addition, cytokines contribute to changes in the tissue environment and interfere with its functioning [17]. By co-culturing senescent cells with normal cells, it was shown that the senescent phenotype could be transmitted to surrounding cells via soluble SASP proteins [104].

The secretory phenotype depends on both the inducing stimulus of senescence and the cell type. Its activity can be regulated by certain proteins of the DNA damage response pathway (DDR pathway) such as ATM and CHK2 [105]. In addition, the expression of many SASP components such as IL-8 or IL-6 is regulated by the activity of the transcription factor NF-κB, responsible for the development of inflammation. It was shown that the down-regulation of a NF-κB subunit, p65, resulted in a reduced level of secreted IL-8 and lower levels of mRNAs encoding IL-8, RANTES and GROα [106]. Inflammation, in particular chronic inflammation, is associated with the pathogenesis of many diseases including those associated with age, for example Alzheimer’s disease, cardiovascular diseases, cancer, diabetes and many others. Curcumin, due to its anti-inflammatory properties, can inhibit NF-κB activity and decrease the level of TNFα, which is the most effective activator of the NF-κB pathway [81,107]. We have shown that short-term cell treatment with low concentrations of curcumin has a positive effect on normal young cells by decreasing the level of secreted pro-inflammatory cytokines. Such treatment decreased IL-8 and VEGF after single application [88]. However, this effect is not observed during the permanent treatment of cells during replicative senescence. Curcumin did not reduce IL-6, IL-8 and VEGF. Quite the reverse: curcumin, due to senescence induction, increased the level of IL-6 and IL-8 [91]. Moreover, low doses of curcumin lead to increased production of sirtuin; i.e., NAD-dependent deacetylases, and sirtuin 1 reduces inflammation by inhibiting NF-κB signaling [108] (see also Section 5.1). To summarize, curcumin, depending on the concentration, is able to reduce or to elevate the level/activity of proteins involved in senescence-associated secretory phenotypes. It is believed that, besides concentration, the impact can be cell-context dependent (the type of stimuli/senescence inductor).

### 5.3. Curcumin and Its Role in Autophagy

The role of autophagy in ageing is evidenced by numerous studies on model organisms from yeast to mice. The expression of proteins involved in autophagy (in particular, those encoded by the ATG gene family) is required for lifespan extension. Moreover, the overexpression of some autophagy proteins is sufficient to prolong lifespan [109]. The key regulators of autophagy are mammalian target of rapamycin (mTOR) kinase and AMP-activated kinase (AMPK). Inhibition of the mTOR pathway and activation of the AMPK pathway extended the lifespan and healthspan of some model organisms [109]. Besides autophagy regulation, these signaling pathways are responsible for nutrient sensing, similarly to the insulin/IGF1 pathway, the inhibition of which was also proven to postpone ageing in animal models [110]. A number of dietary supplements and drugs can stimulate autophagy via the inhibition of mTOR or activation of AMPK pathways; e.g. resveratrol and spermidine or rapamycin and metformin, respectively. However, long-term treatment with metformin can bring about some side effects such as immunosuppression [111]. Curcumin, as mentioned before, regulates the level and activity of AMPK and, as shown in numerous studies, is able to inhibit mTOR level/activity [74,112], which suggests that it can also affect autophagy.

During cancer development, autophagy can be a double-edged sword. At benign stages of the disease, functional autophagy acts as a tumor suppressor by eliminating damaged cells and organelles and by limiting cell proliferation and maintaining genomic stability [113]. In metastasizing highly proliferating cancer cells, functional autophagy delivers much-needed energy and building blocks, thus facilitating undisturbed progression through the cell cycle [114]. Moreover, active autophagy enables cancer cells to overcome the extremely negative influence of the tumor microenvironment, such as hypoxia, inflammation and energy depletion [115]. That is why the inhibition of either induction or autophagy flux in later stages of cancer disease can have a detrimental effect on cancer cells.

Curcumin shows both activating [116] and inhibitory properties regarding autophagy [117]. The action of curcumin is highly dependent on the type of cancer cells [117]. Curcumin can modulate distinct and diverse molecular targets, including Beclin-1 and p53 [118]. Even more interestingly, curcumin can induce a non-apoptotic form of programmed cell death (PCD) called autophagy-associated cell death (type II PCD), which is caspase-independent and does not involve inflammatory response [119]. In summary, curcumin, by its modulatory impact on autophagy, is able to regulate both cancer cell senescence and tumor progression.

The deregulation of the autophagy process is also a culprit in neurodegenerative diseases. Neuronal accumulation of mutated huntingtin or misfolded amyloid beta proteins (Aβ) plays a key role in pathogenesis in Huntington’s and Alzheimer’s diseases, respectively. Curcumin can induce the degradation of misfolded proteins or damaged organelles due to different mechanisms. Firstly, it induces the biogenesis of lysosomes by activating TFEB [120]. Secondly, curcumin restores the physiological level of HSP70, which facilitates proper cargo loading into lysosomes [121]. Furthermore, curcumin induces mitophagy [122], thus lowering oxidative stress, which improves neuronal survival [123].

### 5.4. Curcumin and Cancer

Ageing is one of the factors that promotes cancer development. Among many beneficial effects exerted by curcumin on age-related diseases are its anti-cancer properties. Curcumin was shown to act at different stages of cancer development, starting from cancer initiation to tumor growth and metastasis [124]. There are many molecular targets and signaling pathways that are affected by curcumin. Among them are transcription factors, such as NF-κB and AP1, inflammatory cytokines, growth factors, receptors, kinases and many others [125]. Studies performed in vitro revealed that curcumin induced cell death in many different types of cancer cells [126,127]. Even more, curcumin is able to kill cancer cells that are resistant to commonly-used chemotherapeutic drugs by inducing apoptosis or mitotic catastrophe, as has been revealed by our studies [128,129,130,131,132]. Of note, we have shown that curcumin can induce atypical apoptosis, which is not accompanied either by DNA fragmentation or casapase-3 and -7 activation [132,133,134,135]. The lack of DNA fragmentation in dying cancer cells resulted from the inhibition of DNA fragmentation factor 40 (DFF40), which is a caspase-activated DNA endonuclease [136]. However, it is important to mention that, in these studies, curcumin was used in a relatively high concentration (50 µM).

Both carcinogenesis and ageing are related to increased genomic instability. Those detrimental changes that appear in DNA accumulate in the organism during ageing and favor carcinogenesis. Both increased hypomethylation and accelerated ROS production are observed during ageing. It has been demonstrated that those factors may lead to mutations that appear in protooncogenes and tumor suppressor genes [137]. According to a well-described model of cancer progression, the sequential activation or inactivation of those genes drives cancer development [138]. Importantly, curcumin can potentially decrease the probability of mutations. First of all, curcumin was shown to possess chemopreventive activity. A number of studies have demonstrated that it decreases cancer development induced by certain carcinogens simply by suppressing the mutagenic effect [124]. Curcumin acts also as an antioxidant, and in this way it can protect DNA from mutation [55]. Finally, curcumin modulates the epigenetic landscape by influencing histone acetylation, DNA methylation and miRNA expression [68]. Thus, we can speculate that, thanks to this activity, curcumin may exert anti-ageing and anti-cancer effects, although direct experimental proof is needed.

One of the examples of an oncogene is EGFR. Overexpression and/or mutation of EGFR is characteristic for numerous cancers [139]. The antiproliferative activity of curcumin in cancer cells is based, among others, on the disruption of the EGFR/EGF/TGFα autocrine loop. It inhibits both the phosphorylation of the receptor as well as expression of its ligands [125]. Furthermore, curcumin inhibits downstream signaling from the EGFR, namely PI3K/Akt/mTOR [140] and ERK/MAPK [141] cascades.

Recently, cellular senescence has been recognized as an important outcome of anticancer therapy. In that case, cell senescence results from DNA damage and DNA damage response pathway activation due to chemotherapeutic drug treatment [142,143,144]. Accordingly, we have demonstrated that curcumin at low, non-cytotoxic concentrations induced the senescence of human colon HCT116 cancer cells, MCF-7 human breast cancer cells and U2OS human osteosarcoma cell line [97,145]. The ability of curcumin to cause DNA damage, which is the main trigger of the senescence program, is questionable. There are some reports showing that curcumin is able to induce DNA damage [146,147,148,149,150] while we have revealed that even after treatment with relatively high, cytotoxic concentrations of curcumin, no DNA damage can be identified in cells [94,128,151]. However, disturbed mitosis progression due to improper mitotic spindle formation in curcumin-treated cancer cells has been shown to cause double strand DNA breaks (DSBs) in mitotic chromosomes [152]. Accordingly, we have demonstrated that DSBs remain unrepaired in cells that survived prolonged mitosis, arrest, and then progressed into the subsequent phase of the cell cycle and, finally, underwent senescence. Moreover, the inhibition of the DNA damage response pathway in curcumin-treated cancer cells attenuated senescence and increased the number of proliferating cells [97]. Thus, apart from killing cancer cells, curcumin can exert its anticancer activity also by inducing the permanent growth arrest of cancer cells due to activation of the senescence program.

Currently, the issue of the reversibility of cancer cell senescence has been raised. We have shown that the senescence of cancer cells can be accompanied by polyploidization [100,145,153]. Improper cell division of polyploid cells leads to the regaining of the proliferation potential [100]. It was also demonstrated that cancer cells undergoing senescence acquire the signature of stemness. Therefore, cells that escape senescence, due to some additional mutations, exhibit much higher tumor-initiation potential than those that were never induced to senesce [154]. Thus, the combination of prosenescent anti-cancer therapy together with senolytics seems to be the best and the safest mode of treatment.

### 5.5. Senolytic Activity of Curcumin

Senolytic drugs are compounds which selectively kill senescent cells. The idea to eliminate senescent cells stems from data showing a causative role of senescent cells in age and age-related diseases [3,155]. The accumulation of senescent cells increases with age, and they are found at sites of age-related pathologies [156]. As mentioned before, the selective eradication of senescent cells improved healthspan. There are also studies showing that senolytics can induce apoptosis in senescent cells in vitro [157]. Senotherapy, which aims to alleviate age-related ailments by using senolytics by improving the ability of immune cells to clear senescent cells or by reducing the low-grade inflammation state created by senescent cells is a rapidly developing branch of biogerontology [24]. Among the senolytics described so far are both small molecules, such as dasatinib, navitoclax, A1351852, A155463, and FOXO4-related peptide, and natural compounds such as piperlongumine, quercetin and fisetin [157]. Although curcumin has been shown to exert anti-ageing effects in different experimental approaches, there are only a few data displaying its capacity to modulate cell senescence. Accordingly, curcumin was applied in a mouse model of chemically-induced diabetes mellitus, which is characterized by the impairment of endothelial progenitor cells (EPCs). Results revealed that curcumin application to type I diabetic mice significantly improved blood circulation and increased capillary density in ischemic hind limbs. An in-vitro study also revealed that the angiogenesis, migration, and proliferation abilities of EPCs and the number of senescent EPCs, shown as a number of SA-β-gal-positive cells, returned to the non-pathological level following curcumin application [158].

Cell senescence refers originally to the cell cycle arrest of previously proliferation-competent cells. However, some hallmarks of cell senescence such as increased SA-β-gal activity can be found in non-proliferating neurons in the cell culture and in the brain [22]. Another feature of senescent cells, both previously proliferation-competent and non-dividing post-mitotic cells, such as neurons, is the accumulation of lipofuscin [159]. Thus, it has been revealed that in the CA1 region of rat hippocampus, in which cells were induced to senesce by treatment with d-galactose, curcumin, applied together with piperine, substantially reduced lipofuscin aggregates [160]. Another group, using the same model of rat senescence, has shown that the injection of curcumin lowered p16 mRNA level in premature ovarian failure. However, it is not known whether curcumin induced apoptosis in p16-positive cells, especially whether the overall parameters of apoptosis were increased in the ovaries [161]. Subsequently, in atherosclerotic rats, curcumin diminished SA-β-gal activity in the aorta and the level of an inflammatory marker, MCP-1, in serum [162]. Curcumin increased the survival of rat mesenchymal stem cells and decreased population-doubling time indirectly, which indicates that it can influence replicative senescence [163]. In the culture of mice embryonic fibroblasts derived from prematurely aged mice, curcumin reduced the cell number slightly less than fisetin, which is considered a strong senolytic [164]. Our results have shown that curcumin slightly diminished the survival of vascular smooth muscle cells (VSMCs) undergoing replicative but not stress-induced senescence (manuscript in preparation).

Although, originally, the term senolytic referred to agents that induced apoptosis in normal senescent cells, we are tempted to extend this definition to senescent cancer cells. We express the opinion that the induced senescence of cancer cells is harmful as it can lead to cancer recurrence due to polyploidization/depolypoidization associated with senescence and to cell regrowth [23,100,153]. Thus, it is urgent to search for drugs that would be able to kill senescent cancer cells. To our knowledge, there are no data showing that curcumin is able to selectively kill senescent cancer cells. Also, our own data do not support this possibility (manuscript in preparation). However, senescent cancer cells, as normal senescent cells, are characterized by SASP, which creates a pro-inflammatory microenvironment and reinforces cell senescence [165]. The main activator of SASP is the NF-кB transcription factor [105]. Recently, large Reed–Sternberg cells in Hodgkin’s lymphoma, which display many features of cellular senescence, were shown to have an increased activity of NF-кB. Curcumin is one of the NF-кB inhibitors able to reduce the level of IL-6 secreted by senescent cells [166]. Our own results showed that there were no differences in the cytotoxicity of curcumin and SASP activity between non-senescent HCT116 cells and cells induced to senesce, or between normal replicatively and prematurely senescent VSMC (manuscript in preparation). Overall, results so far obtained by us and others do not unequivocally establish curcumin as a strong senolytic compound. However, it should be noted that no one has proved the universality of senolytic compounds. It seems that they are rather cell-type specific [157]; thus, it cannot be excluded that curcumin’s usefulness as a senolytic compound is still awaiting approval.

### 5.6. Bioaviability and the Microbiome

A large number of controversies arise from the low bioavailability of curcumin. However, the low bioavailability of curcumin does not preclude its usage, as beneficial effects are observed in low doses [167,168,169]. As mentioned before, the poor bioavailability of curcumin is related to weak absorption and rapid elimination from the organism due to the high rate of metabolism. Even more, a higher curcumin concentration could be harmful. In organisms, curcumin is metabolized during transition through the digestive tract (mostly in small intestine) and in liver [170]. The most common products of curcumin metabolism are glucuronides [171,172]. Such conjugates (mono- and diglucuronides) are less active, and therefore the results obtained in cell culture can differ from those obtained in vitro. However, in organisms, there is a lysosomal enzyme responsible for the deconjugation of glucuronides, namely β-glucuronidase, and curcumin glucuronide is one of its substrates [173,174,175]. This protein is expressed in all cell types, with high expression in macrophages, and its activity is highest in the liver. β-glucuronidase activity increases in inflammation [176], and a low-grade inflammatory state is associated with ageing and age-related disease. It can be assumed that local concentrations of curcumin can differ from that detected in serum, as described for other polyphenols in tumor tissue [177]. It cannot be excluded that pro-inflammatory conditions associated with ageing and age-related diseases are responsible for elevated concentrations of non-metabolized curcumin in disease-affected tissue/organs. This, in turn, depending on the type of disease, may lead to adverse effects (e.g., senescence induction in neighboring non-senescent cells). Curcumin glucuronidation is only one of the many factors which affect curcumin bioavailability. The concentration in the serum and tissues depends also on numerous different elements such as low concentration in the food (curcuminoids constitute about 4% of turmeric, and curcumin constitutes 70% of curcuminoids) and interaction with other diet ingredients (the most recognized example is the already mentioned piperine). Curcumin is able to cross the blood–brain barrier (BBB) [178,179], although the permeability is limited [180]. Even though the concentration in brain tissue is lower than in serum, curcumin alleviates neuroinflammation. Nowadays, it is frequently claimed that the real activity of the majority of polyphenols, including curcumin, on the organismal level, is not direct but is mediated by microbiota [181,182]. Moreover, there are data which suggest that intestinal bacteria produce a high amount of β-glucuronidase that can elevate the level of free compounds [183]. This means that microbiota can be responsible for drug metabolism and bioavailability. The microbiome changes over the course of a lifetime [184], and ageing is associated with a reduction of microbial diversity in terms of composition, quality and quantity. It is suggested that healthy ageing correlates with microbiome diversity [185]. Microbiota can modulate certain processes, namely innate immunity, sarcopenia and cognitive dysfunction, adding up to frailty [184]. There is some indication that curcumin is able to modulate gut microbial composition (i.e., biodiversity) [186,187,188]. It can be assumed that, by modulating the microbiome, curcumin may reduce some adverse consequences of ageing, at least those related to frailty. In summary, curcumin, through impacts on the microbiota, might positively influence certain organismal functions, and microbiota, by their ability to metabolize curcumin, can regulate its bioavailability.

## 6. Conclusions

**Figure 1:**
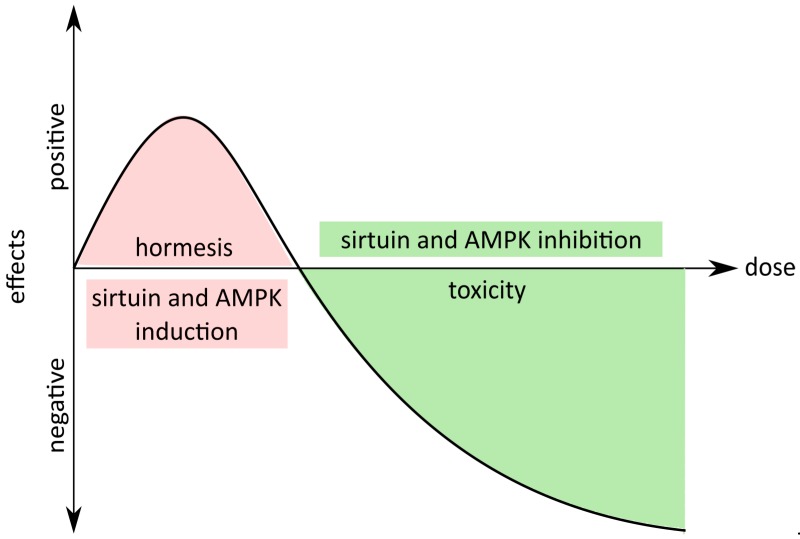
Hormetic properties of curcumin. Low doses of curcumin exert positive effects at the organismal (life extension) and cellular level (activation of sirtuins and AMP-activated kinase (AMPK)); however, at higher doses, curcumin can be toxic or cytostatic (inhibition of sirtuins and AMPK).

Accumulating evidence suggests that one of the causes of organismal ageing is cellular senescence. Senescent cells, besides a loss of proliferating ability, are characterized by SASP, which fuels low-grade chronic inflammation, tumor progression and hinders regeneration. The number of senescent cells increases with age, and their impact on the surrounding cells and the microenvironment escalates. Moreover, senescent cells are found in areas affected by age-related diseases. Studies on genetically modified mice showed that the removal of senescent cells (p16 positive) delayed the onset of ageing, improved tissue functioning, delayed tumorigenesis and allowed animals to reach older age, although without increasing the maximal lifespan. The mice looked younger and were fitter. These data suggest that it may be possible to mitigate age-associated diseases simply by decreasing the number of senescent cells and prompted researchers to look for senolytic drugs and supplements which can selectively induce apoptosis of such cells. Data collected by curcumin researchers showed a large number of beneficial activities for this compound. They mostly concern its anti-cancer activity, but, for several years, the amount of data showing curcumin’s role in the modulation of ageing has been intensively growing, and the issue is widely discussed. Some data suggest a beneficial role, some raise a great deal of skepticism, because unexpected, and sometimes even unfavorable, effects can be observed (e.g., the induction of cell senescence). Curcumin activity depends on the concentration, formulation (pure natural or modified), cell type (differences in vulnerability) and context (organism, disease, manner of administration). Our own data shown that curcumin anti-ageing function results neither from its anti-senescent nor senolytic function, even though curcumin is able to modulate cellular senescence. Low doses activated sirtuins and AMPK, which are considered as having anti-senescent properties, but cytostatic doses inhibited sirtuins and AMPK, inducing, in this manner, cellular senescence (Figure 1).

Moreover, despite a plethora of in vitro as well as in vivo studies on model organisms which document the positive effects of curcumin treatment, human trials are not always as encouraging. For example, preclinical studies on rat models of AD-type sporadic dementia showed that curcumin supplementation was effective in counteracting cognitive decline in animals. However, in clinical studies, after 24 weeks of curcumin supplementation (4 g/day), researchers were unable to detect significant plasma levels of curcumin or any improvement in the cognitive function of AD patients [189]. One of the causes could be the low bioavailability of curcumin, which can be attributed to rapid metabolism and elimination as well as low absorption in the gastro-intestinal tract and mutual interactions with gut microbiota [190].

An excessive increase in bioavailability could be dangerous, especially when curcumin is used as a protective agent. As we have shown, it can induce cellular senescence in relatively low concentrations. This may be beneficial to inhibit tumor progression but could cause also adverse effects, such as the accelerated senescence of different cell types, resulting in premature loss of tissue/organ function. In turn, therapeutic doses should be much higher than protective ones. Moreover, questions arise concerning not only the dose but also when curcumin supplementation should be recommended. Additional meticulous studies are required to solve all doubts and properly exploit such an interesting and promising agent in anti-ageing research.

What is the future of curcumin in anti-ageing strategy? Curcumin possesses a substantial number of supporters and opponents. In our opinion, the positive impact of curcumin on ageing cannot be neglected. Undoubtedly, some precautions in curcumin exploitation necessarily take into account the biphasic response due to its hormetic properties. Curcumin applied in the diet is beneficial. It can act as a tumor suppressor, can lead to the reduction of low-grade inflammation, which is associated with ageing, and to the alleviation of symptoms of age-related diseases, including frailty. Moreover, the impact of curcumin on the microbiome seems to be very promising in the context of the modulation of the ageing process. This issue leaves us with many questions to consider. However, the crucial aspect is its concentration, as stated by Paracelsus, “Omnia sunt venena, nihil est sine veneno. Sola dosis facit venenum”: the dose makes the poison.

**Figure 2:**
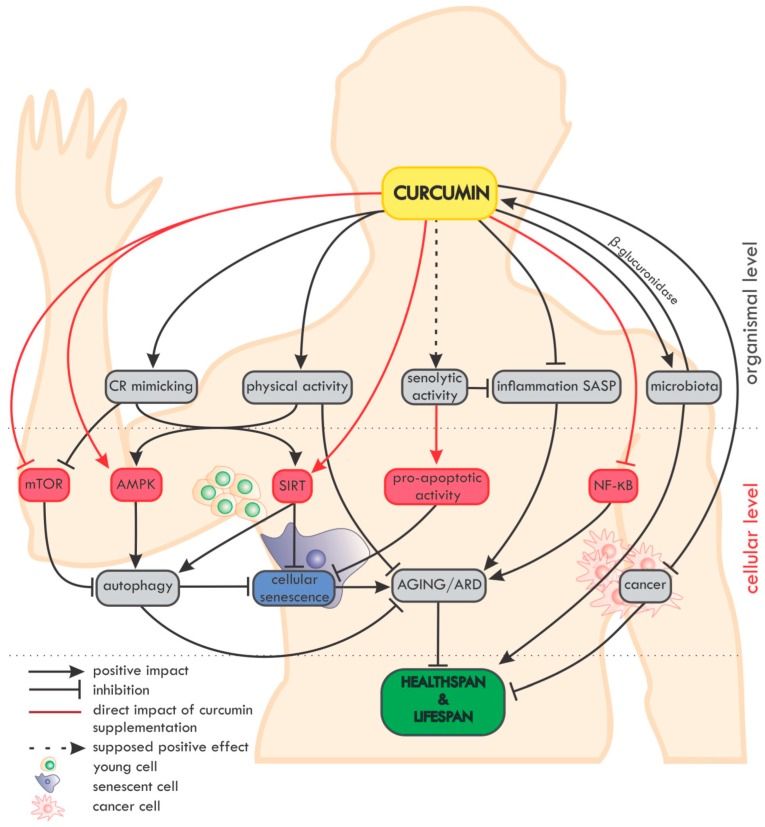
Overview of the impact of curcumin on ageing and age-related diseases (ARD) at the organismal and cellular level. On the organismal level, curcumin mimics caloric restriction (CR) and improves the effectiveness of physical activity (which in fact mimics CR). The potential senolytic activity of curcumin is still unclear, but curcumin can reduce inflammation and SASP, which are also considered as elements of senotherapy. Moreover, curcumin maintains the diversity of the microbiome and, in turn, the microbiota secrete β-glucuronidase, an enzyme, which, by deglucuronisation increases the level of curcumin in tissues. Curcumin is able to protect against cancer and to reduce the progression of already existing tumors. On the cellular level, curcumin elevates the level/activity of some anti-ageing proteins (e.g., sirtuins, AMPK) and inhibits pro-ageing ones (e.g., NF-κB, mTOR). Autophagy, considered as an anti-ageing mechanism, is modulated by curcumin, with the effect of preventing cell senescence. Altogether, by delaying ageing and ARD, curcumin can elongate the healthspan and probably also the lifespan.

An overview of the impact of curcumin on ageing and age-related diseases (ARD) at the organismal and cellular level is summarized in Figure 2.
